# Age-Related Meat Flavor Precursors of Naturally Grazed Sunit Sheep: Metabolomics and Transcriptomics Approaches

**DOI:** 10.3390/foods14091616

**Published:** 2025-05-02

**Authors:** Yajuan Huang, Xige He, Yunfei Han, Lu Chen, Xueting Yu, Jin Li, Xueyan Yun, Rina Sha, Gerelt Borjigin

**Affiliations:** 1 College of Food Science and Engineering, Inner Mongolia Agricultural University, Hohhot 010018, China; 2 China Hohhot Overseas Students Pioneer Park, Hohhot 010018, China; 3 State Key Laboratory of Reproductive Regulation and Breeding of Grassland Livestock, School of Life Sciences, Inner Mongolia University, Hohhot 010020, China; 4 Inner Mongolia Academy of Agricultural and Animal Husbandry Sciences, Hohhot 010031, China

**Keywords:** Sunit sheep, flavor precursor, age, regulation mechanism

## Abstract

This study elucidated the regulatory mechanisms of age-related meat flavor precursors in naturally grazed Sunit sheep of different ages (6, 18, and 30 months) by analyzing their metabolite and mRNA profiles. The longissimus dorsi muscle was sampled from each group and subjected to metabolomics and transcriptomics analyses. A total of 395 differential metabolites (DMs) and 1482 differentially expressed genes (DEGs) were detected across the age groups. As the age increased, the expression levels of *GOT1* and *GLUL* increased, activating arginine biosynthesis and alanine, aspartate, and glutamate metabolism pathways, which promoted the accumulation of umami compounds (L-glutamate and L-glutamine). Meanwhile, the expression level of *LPIN1* increased with age, promoting glycerophospholipid metabolism and contributing to the development of lipid-related aroma. *FADS1* and *FADS2* expressed the highest levels at age Mth_18. This pattern influenced the unsaturated fatty acid biosynthesis pathway and consequently had a regulatory effect on the DHA levels. An amino acid metabolic regulatory network that involved arginine biosynthesis, alanine, aspartate and glutamate metabolisms, and arginine and proline metabolisms was established. This study provided insights into the variations in meat flavor precursors among sheep of different ages and elucidated the underlying regulatory mechanisms.

## 1. Introduction

Meat quality, including the tenderness, flavor, color, and juiciness, influences consumer preferences and choices. Flavor is considered a critical quality characteristic of meat products and plays a pivotal role in determining consumer preferences. Amino acids, fatty acids, nucleotides, and thiamine (VB1) are the basic flavor precursors in raw meat [1]. During cooking, meat flavor develops through a range of reactions, such as the Maillard reaction [2], lipid oxidation [3], vitamin degradation [4], and the interactions between different components and meat flavor precursors. However, meat flavor is affected by a variety of factors, such as breed [5], age [6], gender [7], and rearing practices [8].

Age significantly influences meat flavor. Lee and Kim [6] analyzed the effects of age on the physicochemical characteristics of black goat meat and found that the levels of umami and sweet free amino acids (FAAs) increased with age, whereas bitter FAA levels increased from 9 months of age; the flavor scores improved when the age was less than 9 months, with no significant differences observed thereafter. Chen et al. [9] compared meat flavor precursors and volatile compounds in Chongming white goats of diverse ages and observed that FFAs, triglycerides, and diacylglycerols increased with age. Wang et al. [10] investigated the flavor profiles of Jingyuan sheep at various ages and determined that the hexaldehyde content with a fruit aroma in 6-month-old sheep was significantly higher than those in 2- and 12-month-old sheep. Zhang et al. [11] compared the quality attributes of jerkies produced from New Zealand mutton and lamb. They found that compared with lamb jerky, mutton jerky had slightly lower protein levels and a higher moisture content. During the dry-curing process, proteolysis was influenced by the animal’s age, resulting in significantly higher total FAAs and a different distribution of essential amino acids (EAAs) in mutton compared with lamb. Mutton jerky exhibited more desirable quality characteristics.

Rearing practices are one of the primary factors that influence meat flavor. Noemí et al. [12] investigated the effects of two production systems (intensive and extensive) on the chemical composition and volatile profile of lamb meat, which found that extensively reared lambs produced meat with the highest fat and protein contents. Additionally, extensively raised lamb meat showed higher contents of total acids, alcohols, and esters and a higher concentration of volatile compounds than intensively raised lamb meat. Yang et al. [13] examined the effects of pasture-fed and concentrate-fed diets on the antioxidant activity, meat quality, fatty acid compositions, and volatile compound formation in the longissimus thoracis muscle of Sunit sheep. The results showed that pasture-fed sheep had significantly higher antioxidant enzyme activity and proportions of unsaturated fatty acids, aldehydes, ketones, and furans than concentrate-fed sheep. Wang et al. [8] investigated the effect of different rearing practices, namely, pure pasture grazing, artificial pasture indoor feeding, and indoor feeding, on the lamb flavor. N-acetyl-L-aspartic acid, N-acetyl-L-aspartic acid, N-acetylaspartylglutamate, and L-carnitine levels were higher in the muscles of the pure pasture grazing group compared with those of the pasture indoor feeding and indoor feeding groups. However, the levels of carnosines and creatinine were reduced in the muscles of the pure pasture grazing group. Moreover, glycine, alanine, glutamic acid, and aspartic acid were increased in the muscles of the pure pasture grazing group compared with those of the indoor feeding group.

Sunit sheep are raised on the Xilingol Grassland in Sunit Banner, where the forage species are plentiful, including *Stipa gobicao*, *Stipa breviflora*, and *Allium mongolicum Regel* [13]. In particular, the *Allium mongolicum Regel* can effectively enhance the mutton quality and positively influence the meat flavor characteristics [14]. Local herders typically slaughter sheep at 2-3 years old based on the traditional belief that aging enhances the meat flavor. In our previous research, it was found that the animal’s age significantly impacted the fat deposition [15,16]. We hypothesized that age may affect the accumulation of flavor precursors in meat. In this study, metabolomics and transcriptomics approaches were employed to investigate the effects of age on flavor precursors in the longissimus dorsi (LD) muscle of naturally grazed Sunit sheep. These results help to clarify the expression differences in flavor precursors of the sheep meat of different ages under natural grazing and provide novel insights into studying the mechanism of age-related effects on sheep meat flavor precursors.

## 2. Materials and Methods

### 2.1. Animals and Sample Collection

The experimental materials were sourced from castrated rams (at <30 days post-birth) with uniform genetic backgrounds and rearing conditions. The rams were partitioned into three age groups: 6 months (Mth_6, n = 6, mean weight: 29.23 ± 0.91 kg), 18 months (Mth_18, n = 6, mean weight: 48.67 ± 1.31 kg), and 30 months (Mth_30, n = 6, mean weight: 56.86 ± 1.72 kg). All the rams originated from the same herd in the Xilingol Grassland of Sunit Banner, Inner Mongolia, China (111°54′72.1″ E, 43°35′18″ N, and 900–1400 m asl). These sheep grazed freely on natural grasslands and drank water from 7:00 a.m. to 5:00 p.m. daily. The forage types are described in the Introduction Section. All animals were transported to commercial abattoirs, where they were allowed free access to water and permitted to rest for 12 h. Subsequently, the animals were humanely slaughtered by trained professionals through stunning and exsanguination, in accordance with traditional halal procedures [17]. After slaughter, the longissimus dorsi (LD, 10th to 13th ribs) muscles were sectioned into 3 × 1.5 × 1.5 cm^3^ blocks, rinsed with saline, and placed into labeled lyophilization tubes, which were immediately stored in liquid nitrogen.

### 2.2. Metabolomics Profiling

#### 2.2.1. Sample Preparation and Extraction

The LD samples stored at –80 °C were thawed on ice, minced, and homogenized. Each sample was precisely weighed (20 mg) using a multipoint balance and transferred to a centrifuge tube. The samples were homogenized with a steel ball at 30 Hz for 20 s. Following centrifugation (850× *g* at 4 °C) for 30 s, the pellets were resuspended in 400 μL of a 70% methanol–water internal standard solution, shaken for 5 min, and maintained on ice for 15 min. The samples were centrifuged again (11,300× *g* at 4 °C) for 10 min. A 300 μL aliquot of the supernatant was transferred to a fresh centrifuge tube and incubated at –20 °C for 30 min. Subsequently, the supernatant was subjected to further centrifugation (11,300× *g* at 4 °C) for 3 min, and the resulting supernatant was collected for analysis.

#### 2.2.2. Chromatography Mass Spectrometry Acquisition Conditions

The data acquisition instruments utilized were an ultra-performance liquid chromatograph (UPLC; ExionLC AD) and a tandem mass spectrometer (MS/MS; QTRAP^®^), both sourced from SCIEX, Shanghai, China (https://sciex.com.cn/).

##### T3 and Amide UPLC Conditions

The meat extracts were analyzed using UPLC–electrospray ionization MS/MS (UPLC-ESI-MS/MS). The detailed conditions for the T3 and amide UPLC analyses are provided in Table S1.

##### ESI-QTRAP-MS/MS

The mass spectrometer parameters for the T3 and HILIC analyses were identical. Linear ion trap (LIT) and triple quadrupole (QQQ) scans were acquired using a triple quadrupole–linear ion trap mass spectrometer (QTRAP^®^ LC-MS/MS System), equipped with an ESI Turbo Ion-Spray interface, which was operated in positive and negative ion modes and controlled using Analyst 1.6.3 software (AB Sciex Pet. Ltd., Framingham, MA, USA). The ESI source operation parameters are detailed in Table S2. The collision gas pressure was set to high. The equipment calibration and mass calibration were performed in QQQ and LIT modes in 10 and 100 μmol/L polypropylene glycol solutions, respectively. A specific set of multiple reaction-monitoring transitions for each period was based on the metabolites eluted during the period monitored.

#### 2.2.3. Metabolomics Analysis

The qualitative analysis of metabolites was conducted using an in-house developed target database, MWDB (MetWare Biotechnology Co., Ltd., Wuhan, China), based on the retention time (RT), precursor/product ion pairs, and MS/MS spectral data. Quantitative analysis was performed in multiple reaction monitoring (MRM) mode using a triple quadrupole mass spectrometer. Data processing was carried out with Analyst 1.6.3 software (AB Sciex Pet. Ltd., Framingham, MA, USA). The identified metabolites were annotated against the KEGG database (https://www.genome.jp/kegg/, accessed on 17 November 2022) and HMDB database (https://hmdb.ca/metabolites, accessed on 17 November 2022). Metabolite data were log2-transformed for the statistical analysis to improve the normality. Principal component analysis (PCA) and orthogonal partial least squares discriminant analysis (OPLS-DA), along with orthogonal signal correction, were performed using the MetaboAnalystR package. Significantly regulated metabolic products and their variations between groups were identified based on variable importance in projection (VIP) values derived from the OPLS-DA results. The VIP value represents the impact of the intergroup differences on the classification and discrimination of metabolites in the model. Additionally, the average abundance of the metabolites across the six samples per group was determined, and the fold change (FC) was calculated by comparing the average abundance between the groups. The metabolites with FC ≥ 1.2 or ≤0.83 and VIP > 1 were considered differential metabolites (DMs) [18]. The k-means function was used in the R software (R version 4.2.0) for k-means cluster analysis. The MetaboAnalyst 4.0 (www.metaboanalyst.ca/, accessed on 25 May 2023) was employed for annotation and enrichment analysis. Significantly enriched pathways were identified using the hypergeometric *p*-value for a given list of metabolites.

### 2.3. Transcriptomics Analysis

#### 2.3.1. RNA Extraction, cDNA Preparation, and Sequencing

The TRIzol reagent (Thermo Fisher, Waltham, MA, USA) was employed to isolate the total RNA from the LD samples according to the manufacturer’s instructions. The quantity and purity of the total RNA were assessed using a Bioanalyzer 2100 and RNA 6000 Nano LabChip Kit (Agilent, Santa Clara, CA, USA), with an RNA integrity number (RIN) > 7.0. The Ribo-Zero Gold Kit (Thermo Fisher, Waltham, MA, USA) was employed to remove the rRNA from approximately 5 μg of total RNA according to the manufacturer’s specifications. After the purification, divalent cations were used to fragment poly(A)-tailed RNA into smaller segments at high temperatures. Subsequently, the fragmented RNA was reverse-transcribed to generate the final cDNA library, and the mRNA-seq library preparation was performed according to the Illumina kit protocol (Thermo Fisher, Waltham, MA, USA), generating paired-end libraries with 300 ± 50 bp inserts. Sequencing was subsequently carried out on the NovaSeq 6000 (LC-Bio Technology CO., Ltd., Hangzhou, China) platform following the recommended workflow.

#### 2.3.2. Transcript Assembly

FastQC software (Version 0.11.5, http://www.bioinformatics.babraham.ac.uk/projects/fastqc/, accessed on 12 December 2022) was employed to assess the sequence quality. Cutadapt (v1.9.1) was employed to eliminate adaptor contamination, low-quality bases, and undetermined bases from the raw data. Clean reads were aligned to the sheep genome (Oar_v3.1) using Bowtie2 and Tophat2. The aligned reads were assembled with StringTie. A comprehensive transcriptome was reconstructed by merging all the sheep sample transcriptomes via Perl scripts. Transcript expression levels were estimated using StringTie and the R package Ballgown, and normalized to fragments per kilobase million (FPKM).

#### 2.3.3. Transcriptomics Data Analysis

The differentially expression genes (DEGs) of two comparative combinations (three biological repeats in each group) were examined using the DESeq2 R package (1.16.1). The DEGs were identified based on FC ≥ 1.2 or ≤0.83 and *p* < 0.05 [19]. OmicStudio tools (https://www.omicstudio.cn/tool, accessed on 26 July 2023) and the Kyoto Encyclopedia of Genes and Genomes (KEGG) were used for the enrichment analysis of the DEGs.

#### 2.3.4. Real-Time Quantitative Polymerase Chain Reaction (RT-qPCR) Analysis

To verify the accuracy of the RNA sequencing results, eight DEGs (*AMPD1*, *ANXA1*, *DCN*, *UCP3*, *TRIM63*, *ACACB*, *BAG2*, and *FABP4*) were arbitrarily chosen for RT-qPCR. The total RNA, consistent with the transcriptomics analysis of the LD, was extracted. Subsequently, cDNA was synthesized using the HiScript™ II Q RT SuperMix (Vazyme Biotech Co., Ltd., Nanjing, China) for qPCR (+gDNA wiper) kit. The ChamQ Universal SYBR qPCR Master Mix kit (Vazyme Biotech Co., Ltd., Nanjing, China) was then employed for the amplification of cDNA via qPCR. Dissolution curve analysis was conducted following the procedure provided with the Roche LightCycler^®^96 instrument. To standardize the results, the 2^−ΔΔCT^ method was employed to calculate the relative mRNA expression levels, with β-actin as the internal control [20]. The results are expressed as the mean ± standard error (SE) of three replicates per sample. The primer sequences (Biotech Co. Ltd., Shanghai, China) for the DEGs are listed in Supplementary Table S3.

### 2.4. Integrative Analysis of Metabolomics and Transcriptomics Data

To explore the potential interaction network between the DMs and DEGs in the sheep LD muscle across different ages, an integrative analysis was conducted using metabolomics and transcriptomics data from the LD muscles from the sheep of three different ages. The DMs and DEGs in pathways related to amino acid and lipid metabolisms were selected, and Pearson’s correlation analysis was used to analyze the correlation coefficients between amino acid and lipid-related DMs and DEGs. The threshold of the gene–metabolite correlation was set to |r| > 0.5 and *p* < 0.05. The OmicStudio tools (https://www.omicstudio.cn/tool, accessed on 26 July 2023) and Cytoscape (v3.10.1) were applied to generate DMs and DEGs correlation heatmap and network diagram to explore the interaction between genes and metabolites.

## 3. Results

### 3.1. Metabolomics Analysis

#### 3.1.1. Metabolomics Profiling and OPLS-DA Results

**Figure 1:**
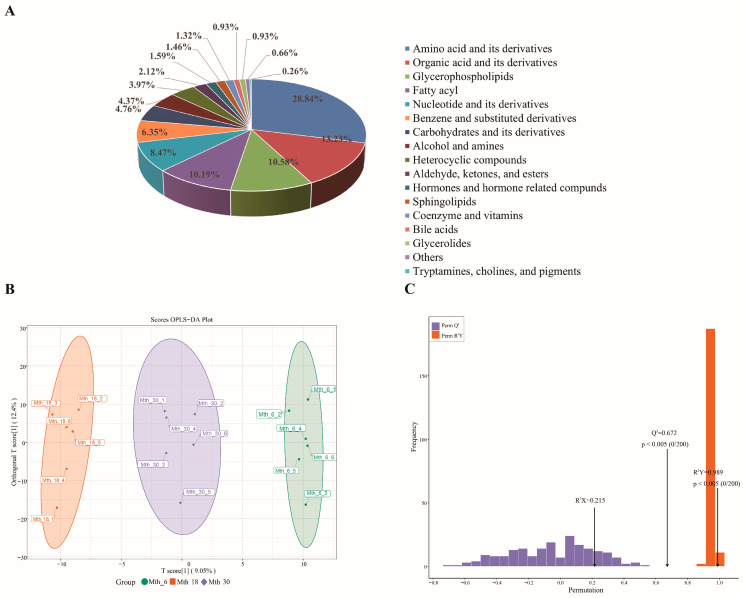
Metabolite categories and OPLS-DA results of Sunit sheep longissimus dorsi (LD) muscles at three ages. (**A**) Pie chart showing the proportion of all metabolite categories. (**B**) OPLS-DA score plots showing the separation at different ages. (**C**) Validation of the OPLS-DA model demonstrating excellent predictive ability (permutation test: R^2^X = 0.215, R^2^Y = 0.989, Q^2^ = 0.672).

In total, 756 metabolites were detected in the LD muscle samples from three age groups and these metabolites were categorized into 17 groups (Figure 1A). The primary flavor-related metabolites were amino acids and their derivatives (28.84%); organic acids and their derivatives (13.23%); glycerophospholipids (GPs, 10.58%); fatty acyls (10.19%); nucleotides and their derivatives (8.47%); benzene and its substituted derivatives (6.35%); carbohydrates and their derivatives (4.76%); and aldehydes, ketones, and esters (2.12%). Collectively, the metabolites in sheep LD muscles were primarily amino acids, lipids, organic acids, and nucleotides, which accounted for 73.68% of all the metabolites and may be the primary factors affecting meat flavor.

The PCA results in Supplementary Figure S1 reveal a clear distribution of samples aged 6, 18, and 30 months within the principal component space. Notably, PCA1 and PCA2 accounted for 14.86% and 11.75% of the variance, respectively, together explaining 26.61% of the total variance. In this distribution, samples from the Mth_6 individuals were predominantly located on the left side of the PCA plot, those from the Mth_18 individuals occupied the central region, and samples from the Mth_30 individuals were predominantly found on the right side. This pattern suggests a natural progression of metabolic changes as the individuals aged, where distinct metabolic profiles emerged for each age group. The OPLS-DA model, as shown in Figure 1B, C, provided additional validation of these findings, with prediction parameter (R^2^Y) and goodness of prediction (Q^2^) values of 0.989 and 0.672, respectively. These results demonstrate clear separation and significant differences between the groups, indicating that the OPLS-DA-derived model exhibited a strong fit and high predictive power, thus being appropriate for further data analysis.

#### 3.1.2. Differential Metabolites (DMs) Analysis

**Figure 2:**
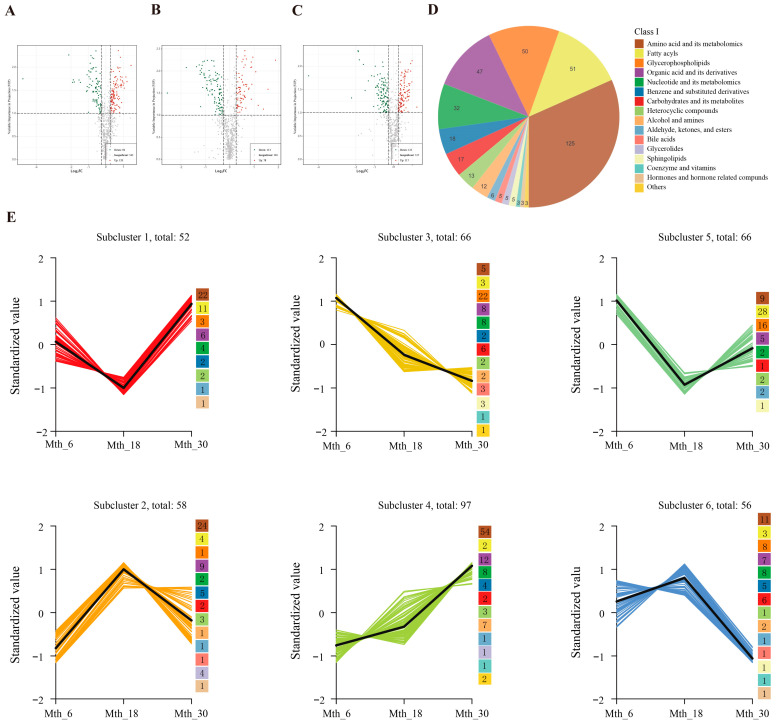
Differential metabolites (DMs) in Sunit sheep LD muscle at different ages. (**A**–**C**) Volcano plots of DMs comparing Mth_18 vs. Mth_6, Mth_30 vs. Mth_18, and Mth_30 vs. Mth_6. (**D**) Pie chart of the classification of 395 DMs. (**E**) K-means clustering of DMs with similar expression patterns. The X-axis indicates the sample groups, and the Y-axis shows the normalized relative metabolite content.

The DMs in each group were further identified and categorized according to the OPLS-DA results. To investigate the shifting trends of the metabolites at different ages, comparisons were made between the Mth_18 vs. Mth_6, Mth_30 vs. Mth_18, and Mth_30 vs. Mth_6 groups (Figure 2). These results were presented in volcano plots and K-means clustering plots. A total of 191 DMs (78 upregulated and 113 downregulated) were identified in the Mth_18 vs. Mth_6 group (Figure 2A). Compared with Mth_6, lipids at Mth_18 showed a downward trend, whereas amino acids, organic acids, and their derivatives exhibited an upward trend. A total of 214 DMs (120 upregulated and 94 downregulated) were identified in the Mth_30 vs. Mth_18 group (Figure 2B). Compared with Mth_18, amino acids, organic acids, and their derivatives, as well as fatty acyl compounds, at Mth_30 showed an upward trend; however, carbohydrates, nucleotides and their derivatives, benzene and substituted derivatives, and GPs exhibited a downward trend. A total of 239 DMs (117 upregulated and 122 downregulated) were identified in the Mth_30 vs. Mth_6 group (Figure 2C). Compared with Mth_6, lipids, carbohydrates, nucleotides, and their derivatives at Mth_30 showed a downward trend, whereas amino acids and their derivatives exhibited an upward trend.

A total of 395 DMs were detected among the three comparison groups, and these DMs were categorized into 16 different categories (Figure 2D). To comprehensively explore the change trend of DMs at different ages, the K-means clustering algorithm was used to cluster 395 annotated metabolites and divided their clustering patterns into six clusters (Figure 2E, Table S4). In clusters 1 and 5, lower expression levels of metabolites were exhibited at Mth_18 compared with those at Mth_6 and Mth_30; the primary components were L-phenylalanine, docosahexaenoic acid (DHA), arachidonic acid (AA), xanthosine, and 2′-deoxyadenosine-5′-monophosphate. In clusters 2 and 6, higher levels of metabolites were expressed at Mth_18 compared with those at Mth_6 and Mth_30; L-glutamic acid, L-citrulline, L-proline, L-serine, L-glutamine, hypoxanthine, cytidine-5′-monophosphate, adenosine 5′-monophosphate (AMP), oleic acid (FFA 18:1), and 5′-deoxy-5′-(methylthio) adenosine were the major components. In cluster 3, the metabolite levels decreased with age, with GPs being the major components. Cluster 4 exhibited a gradual increase with increasing age; L-isoleucine, L-lysine, L-methionine, L-threonine, small peptides, and γ-linolenic acid (FFA 18:3) were the major components.

#### 3.1.3. KEGG Pathway Enrichment Analysis of DMs

**Figure 3:**
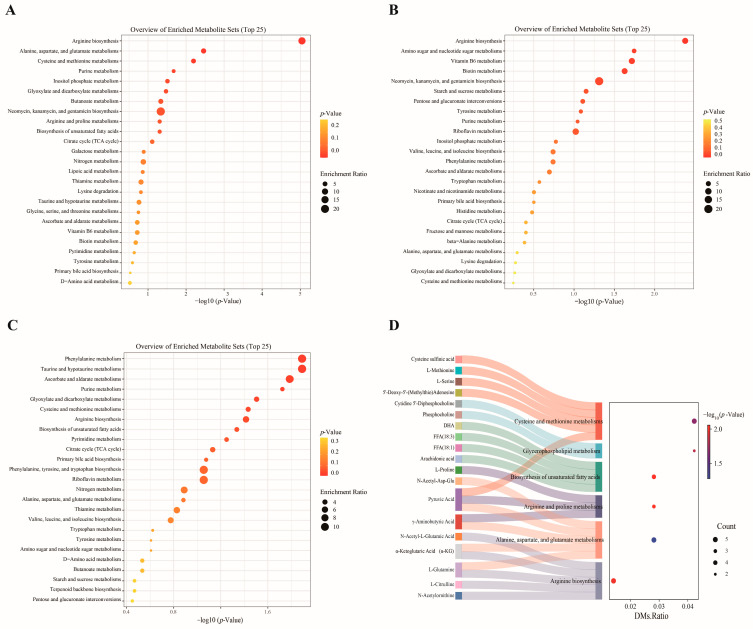
KEGG pathway enrichment profiling of DMs. (**A**–**C**) Pathway enrichment of DMs comparing Mth_18 vs. Mth_6, Mth_30 vs. Mth_18, and Mth_30 vs. Mth_6. (**D**) Key metabolic pathways and metabolites related to flavor precursors. Bubble size indicates impact factor, and color intensity reflects the significance of enrichment.

The top 25 pathways of the three groups are shown in Figure 3A–C. Among these, “arginine biosynthesis”, “alanine, aspartate, and glutamate metabolism”, “arginine and proline metabolism”, “cysteine and methionine metabolism”, “unsaturated fatty acid biosynthesis”, and “glycerophospholipid metabolism” were enriched in two or three comparison groups. The DMs enriched in these pathways included L-glutamine, α-ketoglutaric acid (α-KG), γ-aminobutyric acid, and pyruvic acid (Figure 3D).

### 3.2. Transcriptomics Analysis

#### 3.2.1. Analysis of RNA-Seq and Differential Expression

As shown in Supplementary Table S5, a sum of 65.51 Gbase of raw data was generated from all the samples. After filtering low-quality reads, 64.27 Gbase of valid data was acquired, yielding an average valid read ratio of 98.1%. The average percentages of Q20 and Q30 bases were >99.95% and >97.36%, respectively. The average content of guanine–cytosine was 49.33% of the total nucleobases. Overall, these findings verify the high reliability of the RNA sequencing, allowing for the conduction of follow-up analyses.

**Figure 4:**
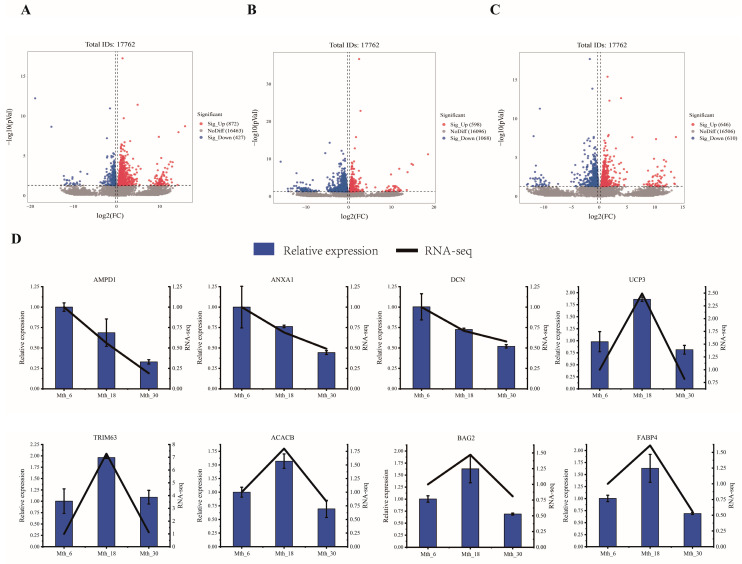
DEGs expression analysis. (**A**–**C**) Volcano plots of DEGs comparing Mth_18 vs. Mth_6, Mth_30 vs. Mth_18, and Mth_30 vs. Mth_6. (**D**) RT-qPCR validation of selected DEGs.

A total of 1482 DEGs were detected across the three comparison groups. Specifically, in Mth_18 vs. Mth_6 (Figure 4A), a total of 1299 DEGs (872 upregulated and 427 downregulated) were identified; in Mth_30 vs. Mth_18 (Figure 4B), a total of 1666 DEGs (598 upregulated, 1068 downregulated) were identified; and in Mth_30 vs. Mth_6 (Figure 4C), a total of 1256 DEGs (646 upregulated, 610 downregulated) were identified.

#### 3.2.2. RT-qPCR Verification of DEGs

The expression patterns aligned with the sequencing data results (Figure 4D), indicating the reliability of the sequencing results.

#### 3.2.3. KEGG Pathway of DEGs

**Figure 5:**
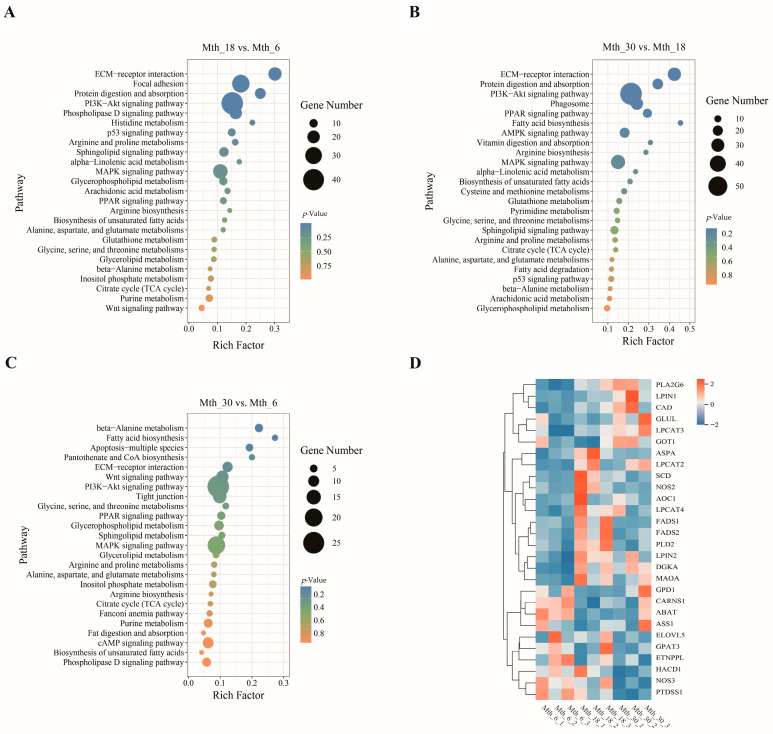
KEGG pathway enrichment analysis of DEGs. (**A**–**C**) Pathway enrichment comparing Mth_18 vs. Mth_6, Mth_30 vs. Mth_18, and Mth_30 vs. Mth_6. (**D**) Heatmap of key genes associated with flavor precursors.

The top 25 pathways were identified in different control groups (Figure 5A–C). KEGG enrichment analysis results revealed that “ECM-receptor interaction”, “PI3K-Akt signaling pathway”, “alanine, aspartate, and glutamate metabolism”, “citrate cycle”, “biosynthesis of unsaturated fatty acids”, “arginine biosynthesis”, “α-linolenic acid metabolism”, “arginine and proline metabolism”, “glycerophospholipid metabolism”, and “MAPK signaling pathway” were the top pathways. The DEGs enriched in these pathways included *COT1*, *GLUL*, *ASS1*, *ASPA*, *CAD*, *ABAT*, *CARNS1*, *AOC1*, *MAOA*, *FADS1*, *SCD*, *PLD2*, *LPIN1*, and *GPD1*. We noticed that the genes *GPD1*, *ABAT*, and *ASS1* were downregulated as the age increased, whereas *PLA2G6*, *LPIN1*, *CAD*, *GLUL*, and *GOT1* were upregulated with increasing age. The expressions of *ASPA*, *LPCAT2*, *SCD*, *FADS1*, *FADS2*, and *AOC1* reached their peak at Mth_18 (Figure 5D).

### 3.3. Integrated Analysis of Metabolomics and Transcriptomics

**Figure 6:**
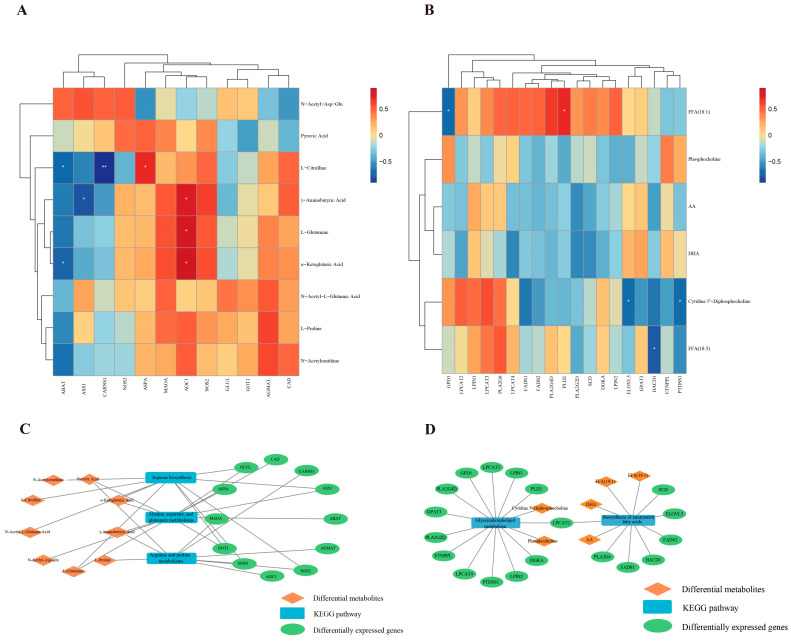
Correlation analysis of DMs and DEGs related to meat flavor precursors. (**A**) Heatmap of correlations between amino acids and DEGs. Red indicates a positive correlation, while blue indicates a negative correlation. (**B**) Heatmap of correlations between lipids and DEGs. Red indicates a positive correlation, while blue indicates a negative correlation. (**C**) Interaction networks of amino acids with DEGs. (**D**) Interaction networks of lipids with DEGs.

**Figure 7:**
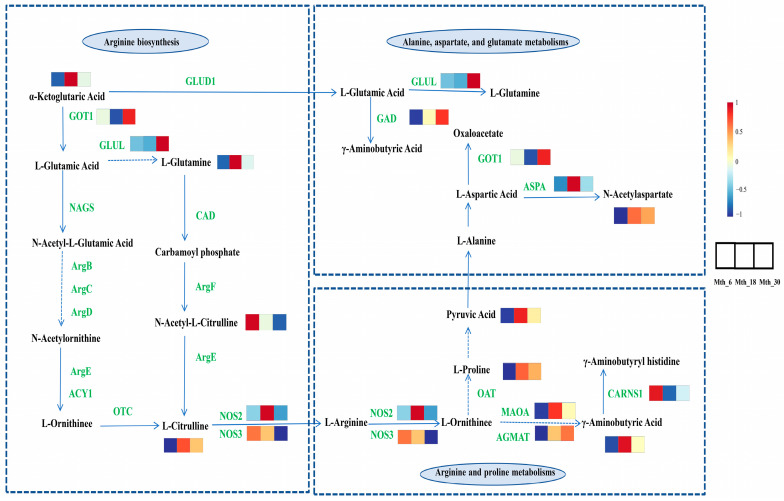
Analysis of key pathways, metabolites, and genes in the regulatory network of amino acid formation in sheep via KEGG pathways.

The DMs and DEGs were co-enriched in the pathways shown in Supplementary Figure S2. These pathways included “arginine biosynthesis”, “alanine, aspartate, and glutamate metabolism”, “arginine and proline metabolism”, “glycerophospholipid metabolism”, “biosynthesis of unsaturated fatty acids”, and “amino sugar and nucleotide sugar metabolism”. These pathways predominantly participated in amino acid and lipid metabolic processes. A correlation analysis was performed between the DMs and DEGs. Twelve DEGs and nine DMs were enriched in pathways related to amino acid metabolism. As shown in Figure 6A, *AOC1* was positively correlated with γ-aminobutyric acid, L-glutamine, and α-KG; *ABAT* was negatively correlated with L-citrulline and α-KG. Nineteen DEGs and six DMs were enriched in pathways related to lipid metabolism. As shown in Figure 6B, *PLD2* was positively correlated with FFA (18:1); *ELOVL5* and *PTDSS1* were negatively correlated with cytidine 5′-diphosphocholine. Cytoscape was employed to construct networks of amino acid and lipid-related genes and metabolites, thereby further elucidating their potential roles (Figure 6C,D). Based on the above results, we constructed a network that may regulate amino acid metabolism (Figure 7), which primarily includes arginine biosynthesis, alanine, aspartate and glutamate metabolisms, and arginine and proline metabolisms. However, the regulatory network underlying the formation of meat flavor precursors is complex, and our understanding is in its infancy. Therefore, further research focused on exploration and confirmation is required.

## 4. Discussion

Flavor precursors in raw meat are crucial for forming meat aroma and flavor, which greatly impact the meat-processing characteristics and consumer choices. Age is a key factor influencing the formation of these flavor precursors. Meanwhile, the metabolism of flavor precursors is precisely regulated by genes.

Amino acids serve as the fundamental building blocks of proteins, and their composition and concentration significantly influence meat flavor characteristics. From Mth_6 to Mth_18, sheep are in a rapid growth phase with a significant increase in protein synthesis to support the quick growth of muscles and other tissues. L-Proline, as one of the key amino acids in meat, exhibits dynamic changes in its intracellular levels that closely correlate with the fluctuating demands of meat production [21]. As sheep age, collagen synthesis is upregulated, and the intracellular L-proline concentration increases significantly. This may be one of the reasons that leads to the upregulation of L-proline in the LD muscle at Mth_18. As sheep age, their physiological demand for amino acids increases—especially L-glutamine [22] and L-serine [23]. From Mth_18 to Mth_30, sheep are leveling off in growth, leading to reduced metabolic demands in muscle tissue; accordingly, the concentrations of L-proline, L-serine, and L-glutamine decrease. Lee and Kim [6] found that during goat growth, the levels of umami and sweet FAAs increased with age, while bitter FAA levels increased from 9 months of age. This result was in conflict with our findings. This discrepancy might stem from the differences in animal breeds and feeding practices. L-citrulline, L-serine, L-threonine, L-lysine, and L-proline are sweet amino acids; L-phenylalanine, L-isoleucine, and L-methionine are bitter amino acids; and L-glutamic acid and L-glutamine are umami amino acids [24]. Notably, amino acids in meat can contribute to its aroma by undergoing a Maillard reaction with reducing sugars during heating [2]. For example, L-methionine, a sulfur-containing amino acid, generates aromatic dimethyl sulfide upon heating, which contributes to the characteristic aroma of meat products [25]. In conclusion, our findings indicate that the flavor-related metabolites in Sunit sheep at Mth_18 and Mth_30 exhibited the greatest complexity.

Similarly, lipids serve as crucial flavor precursors in raw meat and can generate volatile flavor compounds during meat processing [1]. In young animals, the adipose tissue of sheep is in a stage of rapid development, preferentially synthesizing and storing PUFAs, such as DHA and AA, to support their growth and physiological needs [26]. As sheep grow, a decrease in the ability of adipose tissue to synthesize and store PUFAs may occur, which may contribute to the relatively low DHA and AA levels observed at Mth_18. In addition, fatty acid metabolic pathways can change, and adipose tissue may actively break down and release fatty acids, such as FFA (18:1) and FFA (20:3), into the blood circulation and muscle tissues [16]. Wang [27] reported that typical FFA (18:1) derivatives in muscles are heptanal, octanal, and nonanal, which can produce pleasant aromas, such as fruity, fatty, and sweet. Oxidation of n-6 fatty acids (FFA 20:3) and AA produces hexanal, which has a faint grassy aroma [28]. As sheep age, the requirement for n-3 PUFAs increases to support the maintenance of immune function and cell membrane structure. Therefore, metabolic processes in sheep gradually adapt to increase the synthesis and storage of FFA (18:3) to meet these requirements [29]. FFA (18:3) produces a variety of important aromatic compounds, including (E, E)-2,4-decenal, 1-octen-3-ol, 1-octen-3-one, and 2-pentylfuran [30]. This alteration in the lipid profile may be explained by the fact that rapidly growing sheep (Mth_6) prioritize PUFA accumulation for neural and muscular development, while mature grazing animals (Mth_18) transition toward saturated fatty acid (SFA)/monounsaturated fatty acid (MUFA) deposition to meet increased physical activity demands. Glycerophospholipids (GPs) are polar lipids found in all tissues and essential components of cell membranes [16]. GPs primarily include phosphatidylcholine (PC), lysophosphatidylcholine, lysophosphatidylethanolamine, and other common forms [16]. Phospholipids, which have a considerably higher content of unsaturated fatty acids (UFAs) compared with triglycerides, contribute significantly to the generation of meaty aroma compounds during cooking [31]. The GP expression levels decreased with increasing age. This result is consistent with our previous findings [15], which show that glycerophospholipid metabolism was the most active in subcutaneous, perirenal, and tail fat in sheep during the earlier months of age (Mth_6). Notably, the GP expression levels were higher at Mth_6 than at Mth_18 and Mth_30; this may have been because in the early growth stage of sheep, adipose tissue is primarily in the accumulation phase, which requires significant GP synthesis and storage to support the formation and function of cell membranes. As animals age, adipose tissue gradually matures, and its primary function shifts from accumulation to energy storage and release, thus reducing the demand for GP synthesis and storage [16]. At Mth_6, lipid metabolism is significantly enhanced, promoting the generation of various lipid metabolites. At Mth_18, lipid metabolism stabilizes and the concentration of flavor-related metabolites peaks. At Mth_30, the content of flavor metabolites gradually declines with increasing age.

Additionally, nucleotides in meat are also important contributors to its flavor. AMP enhances sweetness, and hypoxanthine can enhance the overall taste of meat [32]. Cytidine-5′-diphosphocholine serves as a crucial biosynthetic precursor for the formation of phosphatidylcholine, a component of cell membranes [33]. Phosphatidylcholine is hydrolyzed during the meat-cooking process, leading to the formation of volatile flavor compounds, such as aldehydes and ketones, which significantly influence the overall sensory profile of meat [34]. The cytidine-5′-diphosphocholine levels increased significantly with age, which may positively affect meat flavor formation by promoting the synthesis of flavor substances and regulating related metabolic pathways. VB1 is a precursor of the essential coenzyme thiamine pyrophosphate required for glucose metabolism. Its degradation produces furan compounds with a strong meaty taste [35]. Zerumbone has a sweet, spicy, rich, and heavy floral aroma [36], which can cooperate with other flavor-enhancing substances during heating to enhance the meat flavor. 2-pentyl-3-phenyl-2-propenal, a member of the cinnamaldehyde class of organic aromatic compounds, has a sweet, floral, and fruity flavor and is used as a flavoring agent [37]. D-mannose enhances the meat flavor by participating in the Maillard reaction to generate flavor compounds, increasing the aroma complexity, balancing flavor components, and protecting the flavor compounds [38]. During meat processing, α-KG can engage in the Maillard reaction to produce a range of flavor compounds, including furans and pyrazines, which enrich the aroma of meat [39]. At Mth_18, the expression levels of the above-mentioned flavor precursors were higher than those at Mth_6 and Mth_30, which may enhance the umami and aroma of the meat, making its flavor more distinctive.

The integration of transcriptomics and metabolomics data reveals key mechanisms of amino acid metabolism in sheep LD muscle (Figure 7). The *GOT* gene is responsible for encoding aspartate aminotransferase. α-KG and L-aspartate are converted to L-glutamate and oxaloacetate by *GOT1* and *GOT2*, the two isoforms of the *GOT* gene [40]. The *GLUL* gene encodes a glutamate–ammonia ligase, which plays a key role in the glutamate–glutamine cycle [41]. The *CAD* gene encodes a multifunctional protein complex comprising carbamoyl-phosphate synthetase 2 (*CPS2*), aspartate transcarbamylase (*ATCase*), and dihydroorotase (*DHOase*). L-glutamine donates an amino group to generate carbamoyl phosphate in a reaction catalyzed by *CAD*. L-ornithine reacts with carbamoyl phosphate to form L-citrulline in a reaction catalyzed by ornithine transcarbamylase [42]. L-citrulline functions as a biological precursor to L-arginine, with most dietary intake being converted to L-arginine through metabolic processes [43]. During arginine and proline metabolism, L-arginine is converted to L-ornithine by L-arginine amidinohydrolase [44]. L-ornithine can be further metabolized to form polyamines, including putrescine, spermidine, and spermine, which are essential for normal cell growth and function. Alternatively, it can be metabolized via a different pathway to produce glutamate [45]. L-ornithine can be converted into L-proline through *OAT* [46]. L-proline undergoes a series of transformations in the metabolic pathway, from arginine to proline and then to pyruvic acid. Pyruvic acid is an important intermediate in glucose metabolism in all biological cells and the interconversion of several substances in the body. In this study, the expression levels of *GOT1* and *GLUL* increased with the age of the sheep, enhancing the levels of L-glutamic acid and L-glutamine. In synergy with *OAT*, this process also promoted the conversion of L-ornithine to L-proline, thereby enhancing the umami flavor of sheep meat.

Glycerophospholipid metabolism, unsaturated fatty acid biosynthesis, and related regulatory genes may be the key factors leading to the differential expression of lipids in sheep muscle. Zhang et al. [30] showed that *LPCAT2* is a key regulator of glysophospholipid metabolism and catalyzes the esterification of glycerophospholipid choline with acyl-coenzyme A to form phosphatidylcholines. *LPIN1* is a member of the lipophospholipase family, which is conserved in mammals. It exhibits phosphohydrolase activity to convert phosphatidic acid to diacylglycerol, which may promote triglyceride and phosphatidylcholine synthesis [47]. *GPD1* is an NAD-dependent dehydrogenase that catalyzes the redox reaction between dihydroxyacetone phosphate and α-glycerol phosphate, converting them to glycerol-3-phosphate. Glycerol-3-phosphate is then used for synthesizing glycerol-backbone lipids [48]. The *LPIN1* levels gradually increased as the sheep aged, which indicates an ascending trend in adipose deposition in conjunction with aging. Hopkins and Mortimer [49] confirmed that older animals exhibited a higher degree of intramuscular fat deposition than their younger counterparts. The presence of oleic acid in muscle and adipose tissues depends on the activity of delta-9 desaturase, which is encoded by the stearoyl-CoA desaturase (*SCD*) gene. This gene promotes de novo lipid synthesis and converts SFAs to MUFAs [50]. In the present study, the *SCD* levels gradually increased as the sheep aged, which acted on the biosynthesis of unsaturated fatty acids and the PPAR signaling pathway. *FADS1* and *FADS2* are drivers that regulate the biosynthetic pathway of long-chain PUFAs (LC-PUFAs). *FADS2*, which encodes delta-6 desaturase, catalyzes the conversion of α-linolenic acid (18:3 n-3) to stearidonic acid (18:4 n-3) and linoleic acid (18:2 n-6) to γ-linolenic acid (18:3 n-6). These intermediates are then further desaturated and elongated to produce eicosapentaenoic acid and DHA. However, *FADS1* encodes delta-5 desaturase, which acts on C20 PUFAs to further desaturate and elongate them into longer-chain LC-PUFAs [51,52]. In this study, the expression levels of *LPIN1* increased with the age of the sheep, indicating an increase in fat deposition, which enhanced the richness and complexity of meat flavor. *FADS1* and *FADS2* negatively correlated with DHA expression and played crucial roles in the synthesis of DHA and AA. These genes are involved in the desaturation and elongation steps of fatty acid metabolism, and their expression levels influence the levels of PUFAs, which are important for flavor development. As the sheep aged, the upregulation of these genes likely contributed to an increase in the PUFA levels, thereby promoting the development of a more complex and desirable flavor profile in the sheep meat.

## 5. Conclusions

This study investigated the accumulation patterns of flavor precursors in naturally grazed Sunit sheep at different ages and revealed the mechanisms that influenced this process. As the age increased, the expression levels of *GOT1* and *GLUL* increased, which promoted the accumulation of umami compounds. *LPIN1* expression also increased, enhancing the development of lipid-related aroma in the meat. Meanwhile, the expression levels of *FADS1* and *FADS2* showed dynamic changes with age, regulating DHA synthesis and thereby influencing the meat flavor. An integrated analysis of metabolic changes and the complexity of flavor precursors across different sheep ages was conducted; the optimal slaughtering stage for flavor in naturally grazed sheep was determined to be from Mth_18 to Mth_30, when the meat’s flavor precursors were the most complex and appealing. This study revealed the molecular basis of flavor formation in naturally grazed sheep. This finding is meaningful to enhance the economic benefits for the sheep meat industry and foster advancements in the field of animal husbandry, as well as to provide future research about investigating the functions of marker genes in specific metabolite in naturally grazed sheep.

## Data Availability

The RNA-seq data were deposited in the NCBI SRA database (PRJNA1231389). All data associated with this study are available by contacting the corresponding authors with a request.

## Supplementary Materials

The following supporting information can be downloaded from https://www.mdpi.com/article/10.3390/foods14091616/s1—Figure S1: Principal component analysis (PCA) results; Figure S2: KEGG analyses of combined metabolomics and transcriptome at different ages; Table S1: T3 and amide UPLC conditions; Table S2: The ESI source operation parameters for the QTRAP^®^ LC-MS/MS system; Table S3: Primer sequences of mRNAs for RT-qPCR; Table S4: K-means clustering results of DMs; Table S5: Summary of mRNA sequencing data.

## Abbreviations

ABAT	4-aminobutyrate aminotransferase
ASS1	argininosuccinate synthase 1
CARNS1	carnosine synthase 1
NOS3	nitric oxide synthase 3
ASPA	aspartoacylase
MAOA	monoamine oxidase A
AOC1	amine oxidase, copper containing 1
GLUL	glutamate–ammonia ligase
GOT1	glutamic-oxaloacetic transaminase 1
AGMAT	agmatinase
CAD	carbamoyl-phosphate synthetase 2
GPAT3	glycerol-3-phosphate acyltransferase 3
PTDSS1	phosphatidylserine synthase
DGKA	diacylglycerol kinase alpha
ETNPPL	diacylglycerol kinase alpha
LPIN1	lipin 1
LPIN2	lipin 2
LPCAT3	lysophosphatidylcholine acyltransferase 3
LPCAT4	lysophosphatidylcholine acyltransferase 4
PLA2G4D	phospholipase A2 group IVD
PLA2G2D	phospholipase A2 group IID
PLD2	phospholipase D2
LPCAT2	lysophosphatidylcholine acyltransferase 2
FADS1	fatty acid desaturase 1
FADS2	fatty acid desaturase 2
HACD1	3-hydroxyacyl-CoA dehydratase 1
SCD	stearoyl-CoA desaturase
PLA2G6	phospholipase A2 group VI
NOS2	nitric oxide synthase 2, aspartate transcarbamylase, and dihydroorotase
