# Supplementary material for: Age-Related Meat Flavor Precursors of Naturally Grazed Sunit Sheep: Metabolomics and Transcriptomics Approaches
Source: Foods. 2025 May 2;14(9):1616. doi: 10.3390/foods14091616 (PMC12071405; doi:10.3390/foods14091616)
Supplement: Supplementary file 1 [file foods-14-01616-s001.zip › Supplementary materials note.pdf]

Fig. S1： Principal component analysis (PCA) results

Fig. S2: KEGG analyses of combined metabolomics and transcriptome at different ages. A: Mth_18 vs. Mth_6; B: Mth_30 vs. Mth_18; C: Mth_30 vs. Mth_6;

Table S1: T3 and amide UPLC conditions;

Table S2: The ESI source operation parameters for the QTRAP® LC-MS/MS system;

Table S3: Primer sequences of mRNAs for RT-qPCR;

Table S4: K-means clustering results of DMs;

Table S5: Summary of mRNA sequencing data.
