# Supplementary material for: Age-Related Meat Flavor Precursors of Naturally Grazed Sunit Sheep: Metabolomics and Transcriptomics Approaches
Source: Foods. 2025 May 2;14(9):1616. doi: 10.3390/foods14091616 (PMC12071405; doi:10.3390/foods14091616)
Supplement: Supplementary file 1 [file foods-14-01616-s001.zip › Supplementary Table S1, S2, S3, S5.pdf]

Table S1. T3 and amide UPLC conditions

Parameter	T3 UPLC Conditions	Amide UPLC Conditions
Chromatographic Column	Waters ACQUITY UPLC HSS T3 C18; 1.8 mm, 2.1 mm × 100 mm	Waters ACQUITY UPLC BEH Amide; 1.7 mm, 2.1 mm × 100 mm
Column Temperature	40 ℃	40 ℃
Flow Rate	0.4 mL/min	0.4 mL/min
Injection Volume	2 μL	2 μL
Solvent System	water with 0.1% formic acid and acetonitrile with 0.1% formic acid	water with 25 mM ammonium formate and 0.4% ammonia acetonitrile
Gradient Program	0 min: 95:5 V/V, 11.0 min: 10:90 V/V, 12.0 min: 10:90 V/V, 12.1 min: 95:5 V/V, 14.0 min: 95:5 V/V	0 min: 10:90 V/V, 9.0 min: 40:60 V/V, 10.0 min: 60:40 V/V, 11.0 min: 60:40 V/V, 11.1 min: 10:90 V/V, 15.0 min: 10:90 V/V

Table S2. The ESI source operation parameters for the QTRAP® LC-MS/MS system

Parameter	Condition
Source Temperature	500 ℃
Ion Spray Voltage	Positive:5500 V;<br>negative–4500 V
Ion Source Gas I	55 psi
Ion Source Gas II	60 psi
Curtain Gas	25.0 psi
Q1 Resolution	Unit, approximately 0.6 Da
Q3 Resolution	Unit, approximately 0.6 Da
Cycle Time	800 ms
Detection Window	40 s

Table S3. Primer sequences of mRNAs for RT-qPCR

Gene Name	Primer Sequence (5'-3')	Product Amplification Length/BP
AMPD1	F: GAGCTACGGGACCTCTACCTGAAG<br>R: TCCACCAAGTCTGCACCTACCTC	92
ANXA1	F: GGCTATGAAGGGTGTTGGAACTCG<br>R: GGATGGCTTGACAGAGAGAGATGC	131
DCN	F: ATCGTCGTAGAACTTGGCACCAAC<br>R: GTCAGCAATGCGGATGTAGGAGAG	99
UCP3	F: CCATCGCCAGGGAGGAAGGG<br>R: TGTCGGTGAGCAGGTGGTAGTC	144
TRIM63	F: GTGGCAGGGAATGACCGTGTG<br>R: TCGCTCTTCTTCTCGTCCAGGATG	146
ACACB	F: CCCTGAACTCCGTCCATTGTAAGC<br>R: CCCTCCTCCTTCCCGATGATGTC	85
BAG2	F: TGTGGAGCAGGAGAAGGAGGTG<br>R: CCATCAGACGGTTCGCAGTCAG	122
FABP4	F: AAGAAGTGGGTGTGGGCTTTGC<br>R: TCCTGGCCCAATTTGAAGGACATC	145
β-actin	F: CCACAGCCGAGCGGGAAATTG<br>R: AGGAGGACGACGCAGCAGTAG	99

Notes: F: Forward, R: Reverse.

Table S5. Summary of mRNA sequencing data

Sample	Raw Data		Valid Data		Valid Ratio (reads)	Q20%	Q30%	GC content%
	Read	Base	Read	Base				
Mth_6_1	50545088	7.58G	49541862	7.43G	98.02	99.94	97.49	48.50
Mth_6_2	43908804	6.59G	43084254	6.46G	98.12	99.95	97.59	49.00
Mth_6_3	53526614	8.03G	52519344	7.88G	98.12	99.94	97.48	49.50
Mth_18_1	47704836	7.16G	46810060	7.02G	98.12	99.95	97.37	49.50
Mth_18_2	45827902	6.87G	44970174	6.75G	98.13	99.95	97.21	49.50
Mth_18_3	51431256	7.71G	50460474	7.57G	98.11	99.95	97.25	49.50
Mth_30_1	49222334	7.38G	48275032	7.24G	98.08	99.95	97.30	49.50
Mth_30_2	45736888	6.86G	44869390	6.73G	98.10	99.95	97.26	49.50
Mth_30_3	48851894	7.33G	47930866	7.19G	98.11	99.95	97.28	49.50
