# Supplementary figures and images for: Age-Related Meat Flavor Precursors of Naturally Grazed Sunit Sheep: Metabolomics and Transcriptomics Approaches
Source: Foods. 2025 May 2;14(9):1616. doi: 10.3390/foods14091616 (PMC12071405; doi:10.3390/foods14091616)

# PCA Analysis

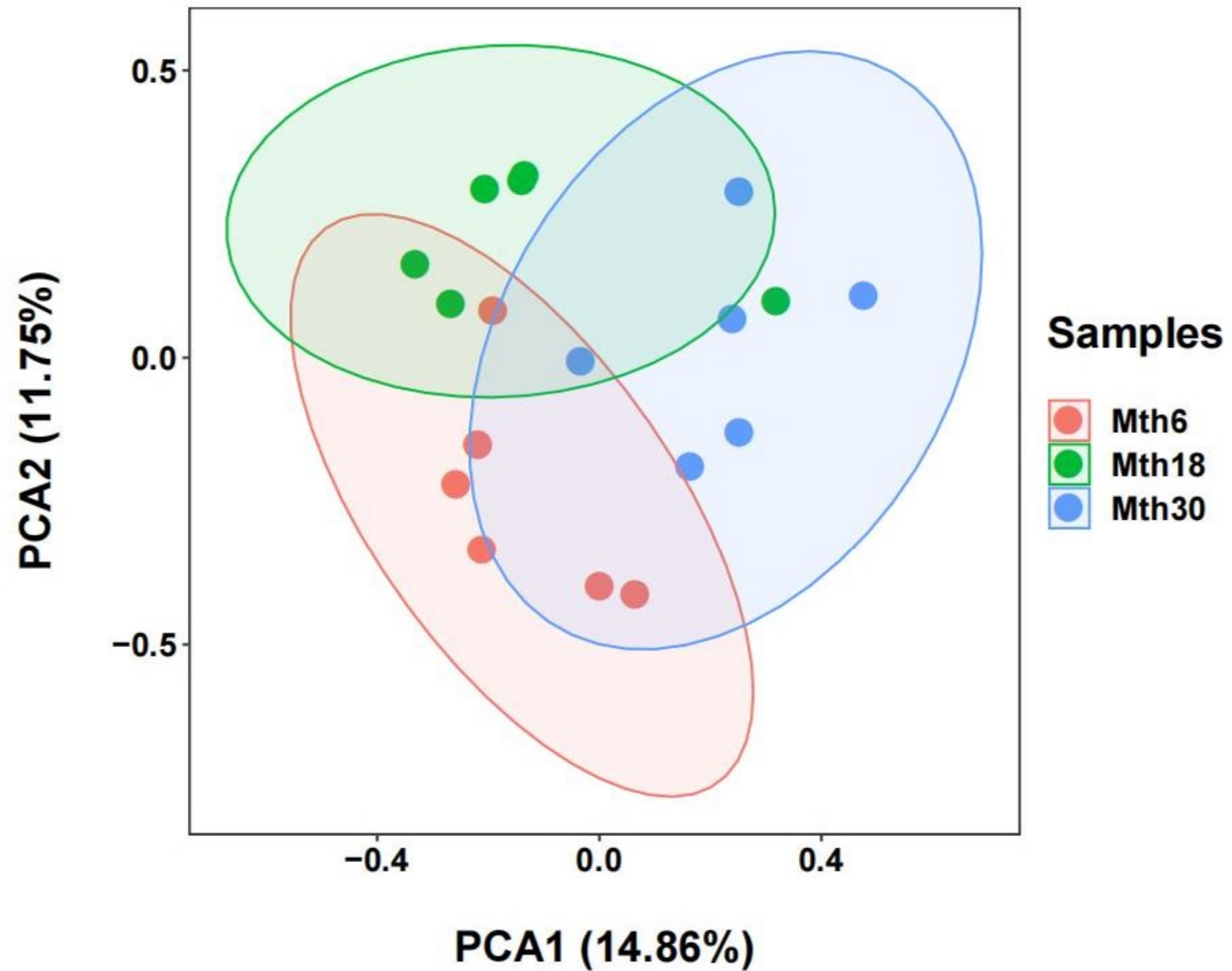

Supplement: Supplementary file 1 [file foods-14-01616-s001.zip › Figure S1ú║Principal component analysis (PCA) results.pdf]

A

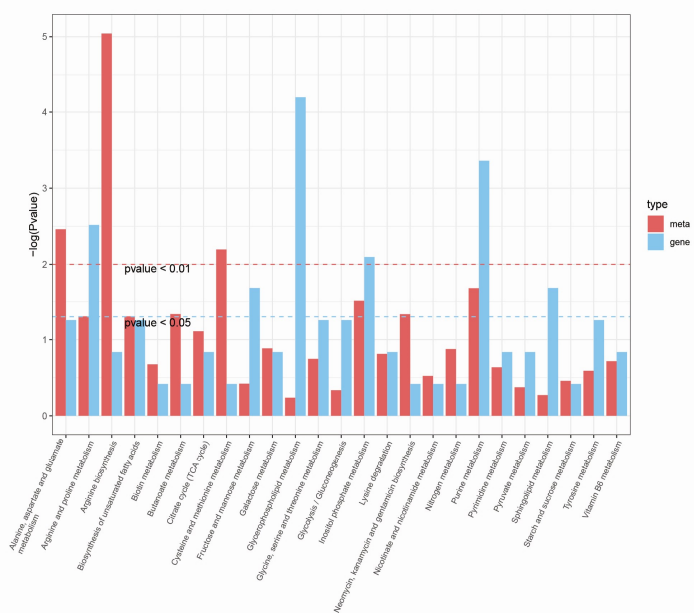

B

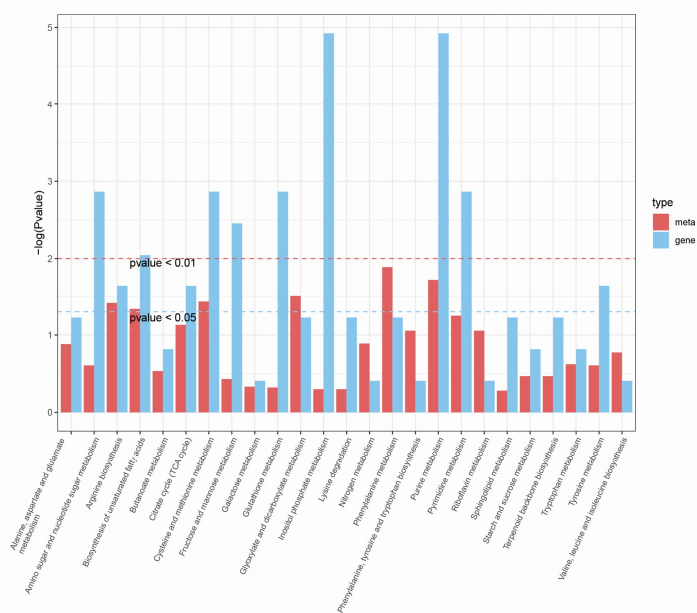

C

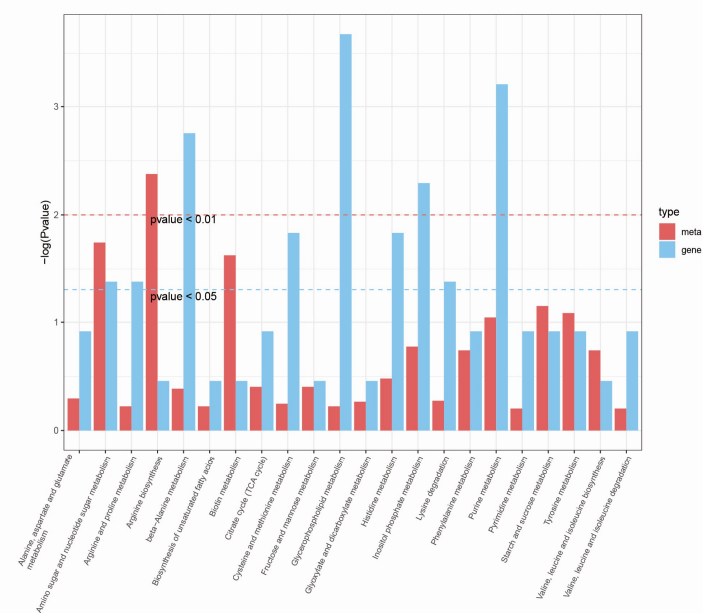

Supplement: Supplementary file 1 [file foods-14-01616-s001.zip › Figure S2ú║ KEGG analyses of combined metabolomics and transcriptome at different ages.pdf]
